# Acetyl-CoA Carboxylases and Diseases

**DOI:** 10.3389/fonc.2022.836058

**Published:** 2022-03-11

**Authors:** Yu Wang, Weixing Yu, Sha Li, Dingyuan Guo, Jie He, Yugang Wang

**Affiliations:** ^1^ Department of Biochemistry and Molecular Biology, School of Basic Medicine, Tongji Medical College, Huazhong University of Science of Technology, Wuhan, China; ^2^ Department of Neurosurgery, Union Hospital, Tongji Medical College, Huazhong University of Science and Technology, Wuhan, China; ^3^ Cell Architecture Research Center, Huazhong University of Science and Technology, Wuhan, China

**Keywords:** acetyl-CoA carboxylase, lipogenesis, cancer metabolism, tumorigenesis, metabolic diseases

## Abstract

Acetyl-CoA carboxylases (ACCs) are enzymes that catalyze the carboxylation of acetyl-CoA to produce malonyl-CoA. In mammals, ACC1 and ACC2 are two members of ACCs. ACC1 localizes in the cytosol and acts as the first and rate-limiting enzyme in the *de novo* fatty acid synthesis pathway. ACC2 localizes on the outer membrane of mitochondria and produces malonyl-CoA to regulate the activity of carnitine palmitoyltransferase 1 (CPT1) that involves in the β-oxidation of fatty acid. Fatty acid synthesis is central in a myriad of physiological and pathological conditions. ACC1 is the major member of ACCs in mammalian, mountains of documents record the roles of ACC1 in various diseases, such as cancer, diabetes, obesity. Besides, acetyl-CoA and malonyl-CoA are cofactors in protein acetylation and malonylation, respectively, so that the manipulation of acetyl-CoA and malonyl-CoA by ACC1 can also markedly influence the profile of protein post-translational modifications, resulting in alternated biological processes in mammalian cells. In the review, we summarize our understandings of ACCs, including their structural features, regulatory mechanisms, and roles in diseases. ACC1 has emerged as a promising target for diseases treatment, so that the specific inhibitors of ACC1 for diseases treatment are also discussed.

## Introduction

In mammalian cells, acetyl-CoA is a global currency that can mediate the carbon transactions between metabolic pathways, including glycolysis, tricarboxylic acid cycle, amino acid metabolism, gluconeogenesis, and fatty acid synthesis. Lipid metabolism or fatty acid metabolism is the bank of acetyl-CoA. It can deposit extra acetyl-CoA in the form of fatty acids and regulate the intracellular availability of acetyl-CoA to the global metabolism network by controlling the conversion of acetyl-CoA into fatty acids. As such, fatty acid synthesis is a central pathway in harnessing a myriad of metabolic pathways and related physiologies in cells.

Acetyl-CoA carboxylases (ACCs) are enzymes that catalyze the carboxylation of acetyl-CoA to produce malonyl-CoA, which in turn is utilized by the fatty acid synthase (FASN) to produce long-chain saturated fatty acids (1). There are two members of ACCs in mammalian cells. ACC1 localizes in the cytosol and takes the major responsibility of converting cytoplasmic acetyl-CoA into malonyl-CoA for fatty acid synthesis (2). Despite ACC2 also catalyzing the conversion of acetyl-CoA into malonyl-CoA, it localizes on the outer membrane of mitochondria that makes the downstream pathways of ACC2-produced malonyl-CoA different from ACC1. It is reported that the ACC2-produced malonyl-CoA can allosterically influence the activity of carnitine palmitoyltransferase 1 (CPT1) in the β-oxidation of fatty acid (3). More functional studies about ACC2 are expected in this field.

Fatty acid synthesis controls the storage and expenditure of carbon source and energy, which can regulate the activities of other metabolic pathways, such as amino acid metabolism and glucose metabolism, so that fatty acid synthesis is frequently alternated to harness the intracellular metabolism network to meet the requirement of materials and energy for diseases progressions, such as cancer and metabolic diseases (4–8). ACC1 is the first rate-limiting enzyme in the fatty acid synthesis that plays a central role in fatty acid synthesis, so that ACC1 is the hub of the fatty acid synthesis-related metabolism network. Its dysregulation in diseases is intensively studied, including the roles of ACC1 in regulating tumour cell proliferation, migration, and metabolic disease progression (9–12). In addition, because acetyl-CoA and malonyl-CoA are cofactors in protein acetylation and malonylation, respectively, the emerging non-metabolic functions of ACC1 in diseases are discussed in recent studies (11, 13, 14), which further expand the roles of ACC1 in physiologies and pathophysiologies. ACC1 is therefore considered as a promising therapeutic target for treating diseases, such as cancer and metabolic diseases.

This review summarizes our current knowledge about ACCs, including the structure of ACCs, the regulatory mechanisms, and the roles of ACCs in tumorigenesis and metabolic diseases. Besides, we briefly introduce ACCs inhibitors that are under investigation for cancer and metabolic diseases therapy.

## Structure of Acetyl-CoA Carboxylases

In mammals, ACCs have two isoforms: ACC1 and ACC2. Human ACC1 (ACCα, 265 kDa) is encoded by the *ACACA* gene on chromosome 17q12 while ACC2 (ACCβ, 275 kDa) is encoded by the *ACACB* gene on chromosome 12q23 (15). ACC1 and ACC2 share 75% identity in amino acid sequence and are composed of conservative domains for enzyme activity (16, 17).

**Figure 1:**
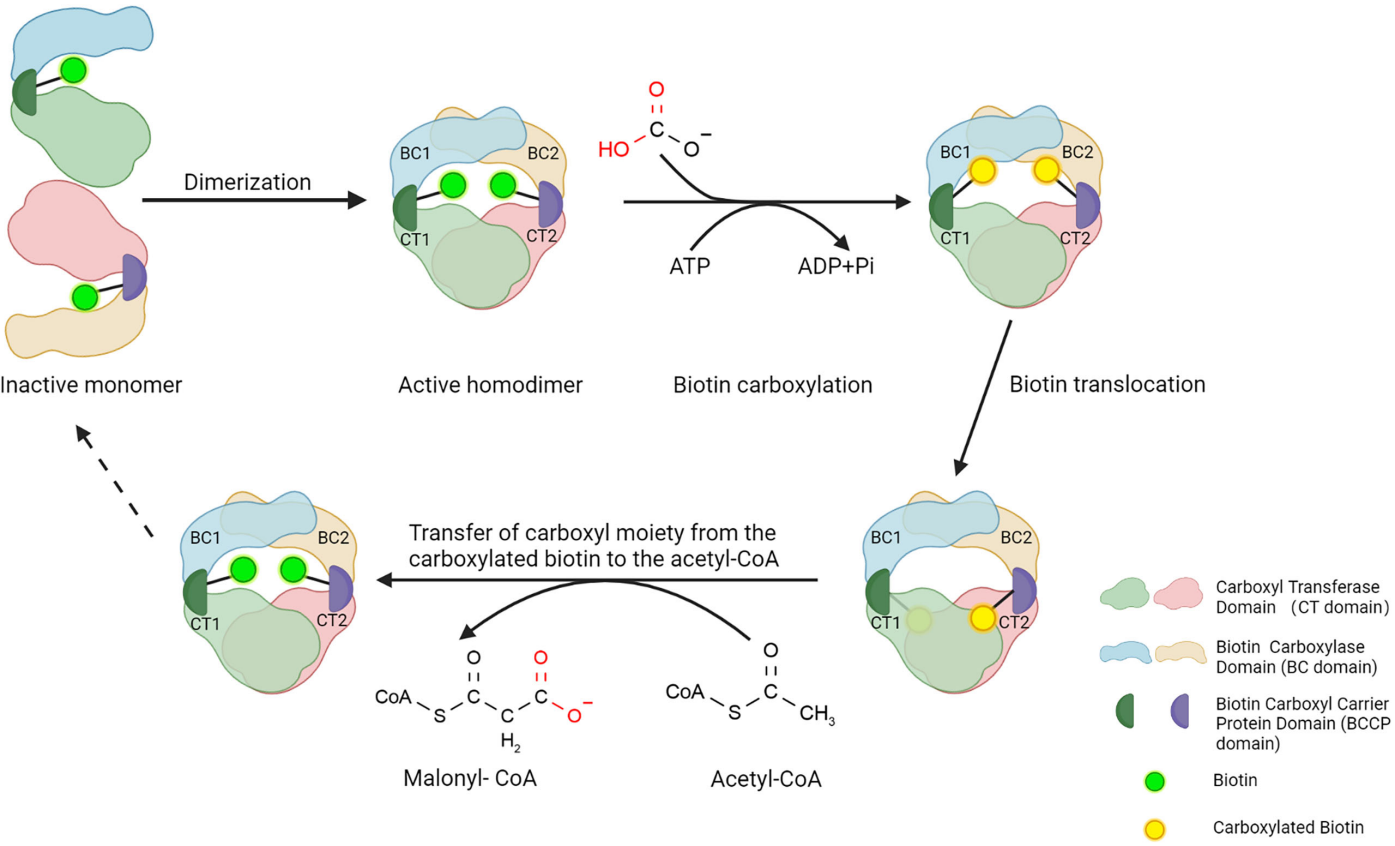
Structure of ACC1 and function of three main domains. Three steps to a functional ACC1: First, the BC domain consumes ATP and catalyzes the carboxylation of biotin, in which bicarbonate serves as the donor of the carboxyl moiety. Subsequently, the BCCP domain transfers the bicarbonate moiety from carboxylated biotin to the CT domain of ACC1. Lastly, the CT domain catalyzes the carboxylation of acetyl-CoA carboxyl moiety, converting acetyl-CoA into malonyl-CoA. Created with BioRender.com.

ACC1 and ACC2 have similar structures and molecule bases in catalyzing carboxylation of acetyl-CoA to produce malonyl-CoA. ACC1 is discussed here in terms of ACCs’ structure. ACC1 contains three major functional domains: a biotin carboxylase domain (BC domain), a carboxyl transferase domain (CT domain), and a biotin carboxyl carrier protein domain (BCCP domain) that links the BC domain and CT domain (**Figure 1**). To perform the catalytic activity, the BC domain of ACC1 firstly consumes ATP and bicarbonate to catalyze the carboxylation of biotin, in which bicarbonate serves as the donor of the carboxyl moiety (18). Then, the BCCP domain transfers the carboxyl moiety from the carboxylated biotin to the CT domain (19), where the carboxyl moiety is transferred to the acetyl-CoA to accomplish the carboxylation of acetyl-CoA, converting acetyl-CoA into malonyl-CoA (20) (**Figure 1**). Although the BC domain and CT domain are linked by the BCCP domain in a single ACC1 molecule, the spatial dimension of ACC1 is so broad that the functional domains are spatially separated, which makes the carboxylated biotin in the BC domain can hardly reach to the acetyl-CoA that bond by the CT domain of the same molecule of ACC1. To link the cascade reactions of acetyl-CoA carboxylation, ACC1 molecules form homodimers to enable the carboxylated biotin in the BC domain reaching to the acetyl-CoA in the CT domain of the other ACC1 molecule of the homodimer (19–23). Therefore, regulating the formation of ACC1 homodimers is considered as an important mechanism controlling the acetyl-CoA carboxylation activity of ACC1.

## Distribution and Functions of Acetyl-CoA Carboxylases

**Figure 2:**
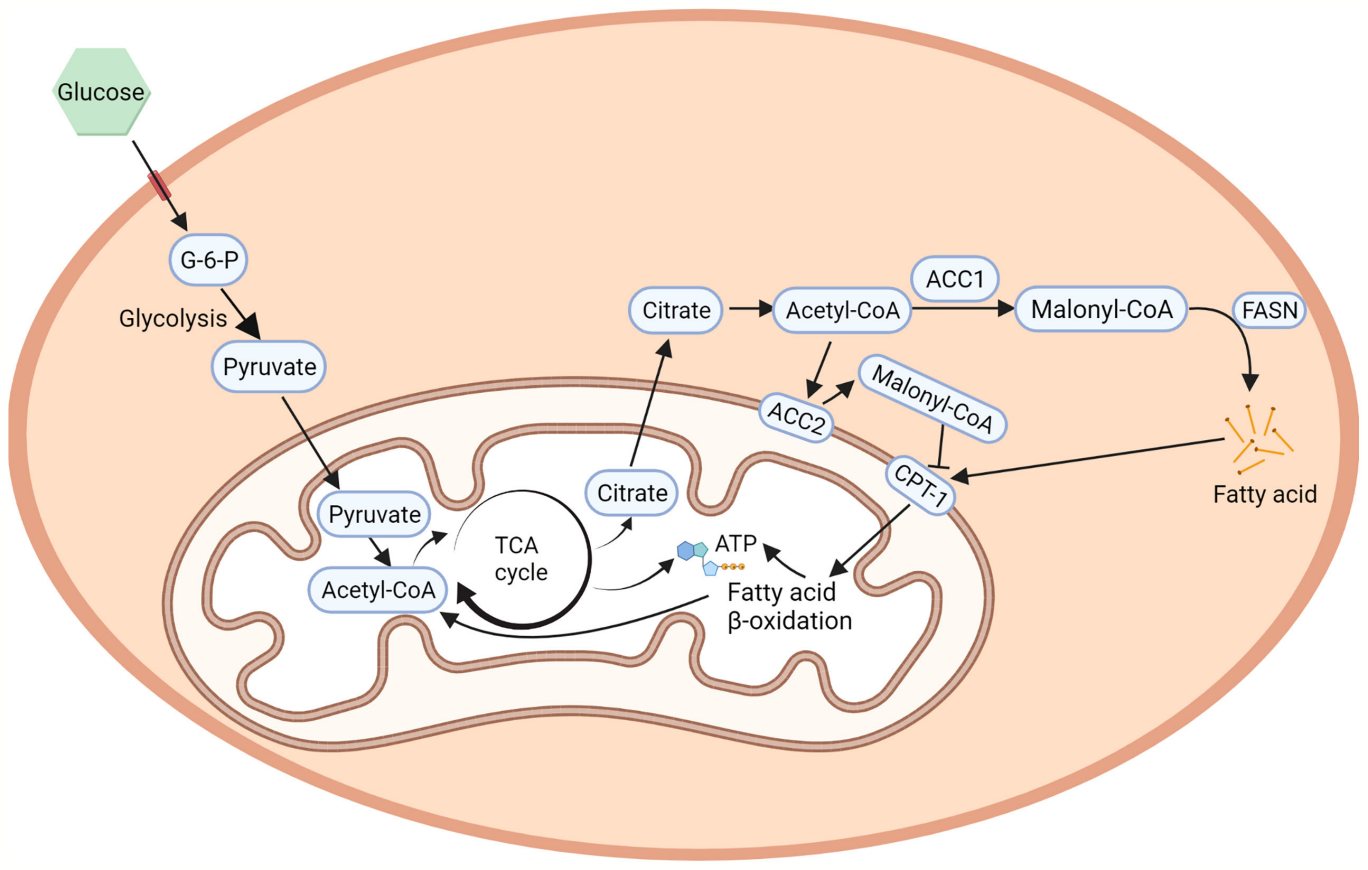
ACCs in fatty acid metabolism. ACC1 is a cytoplasmic protein that catalyzes the conversing of acetyl-CoA to malonyl-CoA in the *de novo* fatty acid biosynthesis (2). On the other hand, the hydrophobicity of the N-terminal region of ACC2 allows its localization to the outer membrane of the mitochondria, regulates CPT1 which controls fatty acid β-oxidation (25). Created with BioRender.com.

ACC1 and ACC2 are widely distributed in organs and tissues in mammals. ACC1 is highly enriched in lipogenic tissues, such as liver and adipose tissue, while ACC2 is majorly expressed in oxidative tissues, such as cardiac and skeletal muscle (24), which are consistent with the functions of ACC1 in lipogenesis and ACC2 in regulating fatty acid β-oxidation. In mammalian cells, ACC1 and ACC2 are differently distributed (**Figure 2**). ACC1 is a cytoplasmic protein that catalyzes the conversion of acetyl-CoA into malonyl-CoA, which is utilized by the fatty acid synthetase (FASN) and subjected to the *de novo* fatty acid biosynthesis (2). It controls the synthesis of mid-and long-chain fatty acids that serve as building blocks for the cell biology process. Inhibiting ACC1 by 5-tetradecyloxy-2-furoic acid (TOFA) can completely inhibit hepatic *de novo* lipogenesis (DNL), which is considered a new strategy for non-alcoholic fatty liver disease (NAFLD) treatment (25). Soraphen A, another ACC1 inhibitor, can pharmacologically inhibit fatty acid synthesis in diet-induced obesity mice and significantly suppress weight gain, which sheds new light on controlling diet-induced obesity (26). Liver-specific ACC1 knockout (LACC1 KO) mice can survive but show dysregulated lipid metabolism and deficiency in triglycerides metabolism (27). In cancer cells, inhibition of ACC1 by Soraphen A significantly reduces saturated and mono-unsaturated phospholipid species and increases the proportion of poly-unsaturation, rendering cells vulnerable to oxidative stresses (28). Moreover, activity-impeded ACC1 reduces cytoplasmic membrane fluidity and impairs mobilities of transmembrane receptors, ultimately impairing cell membrane-dependent biological processes (29).

ACC2 contains a hydrophobic N-terminal region that leads ACC2 attaching to the outer membrane of mitochondria (25). The mitochondria-located ACC2 also catalyzes the conversion of acetyl-CoA into malonyl-CoA. However, instead of entering the *de novo* fatty acid biosynthesis, the ACC2-generated malonyl-CoA locally interacts with carnitine palmitoyltransferase 1 (CPT1) that also localizes on the outer mitochondrial membrane. CPT1 accounts for the first step of long-chain fatty acids β-oxidation in mitochondria. The binding of malonyl-CoA allosterically inhibits the activity of CPT1 and therefore influences the process of fatty acid β-oxidation in mitochondria (30). In animal experiments, inhibition of ACC2 can increase hepatic fat oxidation, reduce hepatic lipids, and improve hepatic insulin sensitivity in mice with NAFLD (31), which is further confirmed in mice with genetic depletion of ACC2 (3, 32, 33). In addition, the fatty acid oxidation rate in the soleus muscle of the ACC2-/- mice is 30% higher than that of wild-type mice and is not affected by the addition of insulin, leading to reduction of body weight under normal food intake and slower weight-gain with high-fat/high-carbohydrate diets (34).

In addition to the roles in metabolic flow, fatty acids, acetyl-CoA, and malonyl-CoA are effector molecules that participate in signaling pathways in cells (35–37). Correspondingly, ACCs, as the consumer of acetyl-CoA and the producer of malonyl-CoA that function as the rate-limiting enzyme in fatty acid synthesis, play intriguing roles in regulating cellular signaling networks. For example, polyunsaturated fatty acids (PUFAs) are the precursors of various signaling molecules, such as eicosanoids, that regulate the activity of sterol-regulatory-element-binding protein 1c (SREBP1c) in fatty acid metabolism in liver (38). Inhibition of ACCs is considered as a promising strategy for treating liver diseases (39). However, on the other hand, it leads to a decrease in malonyl-CoA and the synthesis of downstream PUFA, which in turn activates SREBP1c and upregulates the expression of glycerol-3-phosphate acyltransferase 1 (GPAT1) that catalyzes triglyceride synthesis, stimulating hepatic VLDL secretion and leading to hypertriglyceridemia (40). As such, hypertriglyceridemia is used to be accompanied with the ACCs-targeting therapies. Acetyl-CoA is another instance. It can regulate gene transcription by donating the acetyl-moiety in the acetyltransferases-mediated histone acetylation (41). Inhibition of ACCs can increase the intracellular acetyl-CoA level and stimulate the influx of calcium into the cells, which lead to the activation and translocation of NFAT (nuclear factor of activated T cells 1) into the nucleus to promote the transcription of adhesion and migration-related genes, promoting adhesion and migration of glioblastoma cells through Ca^2+^– NFAT signaling. Malonyl-CoA plays roles in regulating dietary behavior and appetite (42). It is shown that mammalian neural tissue was able to rapidly convert administered acetate into acetyl-CoA, which subsequently entered the Krebs cycle to promote ATP production. Excessive ATP level, in turn, down-regulates AMPK activity and secures ACC2’s enzymatic activity. As a result, malonyl-CoA was produced to a great extent, causing the downstream effector molecular pro-opiomelanocortin upregulation and neuropeptide Y downregulation, eventually leading to loss of appetite in mice (43, 44).

In addition to the metabolic functions, ACC1 and ACC2 regulate protein acetylation by manipulating the availability of acetyl-CoA in cells. In liver-specific ACC1 knockout mice, the amount of acetyl-CoA in the extra-mitochondrial space is substantially elevated, which can serve as the substrate cofactor for acetyltransferases and increase the acetylation of proteins to regulate the functionome, including metabolic enzymes that regulate the metabolism network in ACC1 KO mice (13). Another study also shows that attenuated expression of ACC1 leads to a substantial increase in histone acetylation and alters transcriptional regulation, resulting in increased histone acetylation that consequently regulates biological processes in cells *via* influencing gene transcription (14). While the causal relationship between ACCs’ activities and protein acetylation is confirmed, the detailed mechanism underlying ACC1-related hyperacetylation remains elusive. Besides, ACC1 regulating protein acetylation by controlling the intracellular concentration of acetyl-CoA might also play a role in disease development. A supportive study reported that ACC inhibition regulates smad2 acetylation, which consequently affects the activity of smad2 and breast cancer metastasis (11). Malonyl-CoA is the product of ACCs’ enzymatic reactions. It can also serve as the substrate cofactor in the enzyme-catalyzed protein malonylation. Increased intracellular malonyl-CoA can result in upregulation of protein malonylation, which might affect protein functions and biological activities in cells (9). Despite evidences are supporting the importance of the non-metabolic functions of ACC1 in regulating protein modifications and functions, it is premature to conclude the non-metabolic functions of ACC1 in diseases development and treatment.

Altogether, ACCs regulate the physiologies and pathophysiological processes of cells by executing metabolic and non-metabolic functions. It should sense the alternatives in cells and precisely translate the alternated signals into the responses of cells. As such, sophisticated regulation of ACCs is required to secure the metabolism network matching the physiologies of cells.

## Regulation of Acetyl-CoA Carboxylases

The activities of ACCs in cells can be transcriptionally and post-transcriptionally regulated that are tightly associated with the metabolic status of cells. In general, the protein level and enzymatic activities of ACCs are upregulated in nutrient and energy abundant conditions, aiming to store the excess nutrient and energy in the form of fatty acids. Correspondingly, the protein level and enzymatic activities of ACCs are suppressed in nutrient and energy-deficient conditions, aiming to secure the limited energy and nutrients being utilized for survival (45, 46). The AMP-activated protein kinase (AMPK) is the most studied energy sensor that senses the nutrient and energy status of cells and is an important regulator of ACC1. When cells suffering metabolic stresses, such as glucose deprivation or hypoxia, AMPK is activated that can phosphorylate the serine-79 residues in ACC1 (equivalent to ACC2 Ser212) (47). Phosphorylation of the Ser-79 residue effectively blocks the formation of ACC1 homodimer, leaving ACC1 molecules as monomers that are unable to catalyze acetyl-CoA carboxylation (21). The fatty acid synthesis pathway is therefore suppressed. However, when cells return to a nutrient and energy-abundant environment, the phosphorylation of Ser-79 in ACC1 can be removed by the type 2A protein phosphatase (PP2A), allowing the reformation of ACC1 homodimer that is active in catalyzing acetyl-CoA carboxylation (48, 49). Besides nutrient and energy stresses, the Ser-79 residue in ACC1 can be phosphorylated and maintained to prevent lipogenesis in certain pathophysiological processes. For example, the susceptibility gene 1 (BRCA1) C-terminal (BRCT) domain binds to p-ACC1 to from BRCA1/p-ACC1 complex (50), which prevents dephosphorylation of p-ACC1 and constantly suppress the activity of ACC1 to reprogram the metabolism network in breast cancer. Insulin-like growth factor-1 (IGF-1) treatment can disrupt the interaction between BRCA1 and p-ACC1, which leads to the dephosphorylation and reactivation of ACC1 (51).

In addition to phosphorylation, metabolites that are associated with changes in metabolism can allosterically regulate the activities of ACCs. For example, citrate is an intermediate metabolite in the TCA cycle that can allosterically activate ACC1 to drive the fatty acid synthesis pathway in normal condition (52, 53). Intriguingly, opposite effects of citrate on ACCs’ activities were reported (54), the underlying mechanism remains elusive. Glutamate can allosterically activate phosphatase that mediates dephosphorylation and activation of ACCs in cardiomyocytes, which may contribute to the cardioprotective effects of glutamine against lipolysis (55).. Fatty acyl-CoAs can induce the de-dimerization of ACC1 that inhibits the activities of ACC1 and fatty acid synthesis in cells (54). By interplay with metabolites from different metabolic pathways, ACCs mediate the cross-talk between fatty acid synthesis and other metabolic pathways, forming a sophisticated regulation network to secure the metabolic status fit the physiologies of cells.

The protein levels of ACC1 and ACC2 are dynamically regulated in cells. The expression level of ACC1 can be regulated by certain transcription factors. SREBP1c is a well-studied instance. The *ACACA* gene has two distinct SREBP binding sites, which recruit SREBP1c to initiate RNA Polymerase II-dependent transcription. Carbohydrate response element (ChRE) -binding protein (ChREBP) is another transcription factor that regulates ACC1. It binds to the promoter regions and activates the transcription of *ACACA*, in response to the high-carbohydrate diet (56, 57). Besides transcription, the protein stability of ACC1 can also be regulated. In breast cancer, small interfering RNA-mediated Aldo-keto reductase family 1B10 (AKR1B10) knockdown induces ACC1 degradation *via* the ubiquitination-proteasome pathway, resulting in a markedly drop in fatty acid synthesis in RAO-3 cells (58). In prostate cancer, the expression of prolyl isomerase Pin1 positively correlates with the protein level of ACC1. It binds to ACC1 to prevent ACC1 from entering the lysosomal pathway, leading to the stabilization of ACC1 protein and resulting in enhanced activity of ACC1 in cells (59).

ACC1 activity can also be regulated by post-transcriptional and translational mechanisms. There are 64 exons included in the gene *ACACA* that result in 7 alternatively spliced minor exons (1A, 1B, 1C, 3, 5A’, 5A, and 5B). The exon 5B can lead to transcriptional termination of the upstream exon 5 in two different transcripts, producing a short peptide that leads to the production of truncated ACC1 that affects the transcriptional efficiency and activity of ACC1. These studies suggest that ACC1 activity can be regulated by post-transcriptional and translational mechanisms and consequently result in suppression of fatty acid synthesis (60). The protein level of ACC1 can be post-transcriptionally reduced in calcium/calmodulin-dependent protein kinase kinase2 (CAMKK2) knock out cells, suppressing the proliferation of human prostate cancer cells (61).

Taken together, ACC1 and ACC2 are sophisticatedly regulated in cells to make the process of fatty acid synthesis, as well as its cross-talk metabolic networks, meet the physiologies of cells. There are myriad factors that regulate ACC1’s activities, including nutrients, protein kinases, phosphatases, allosteric regulators, transcriptional factors et al. Dysregulation of these regulatory factors usually serves as causative signaling for the development of cancer and metabolic diseases (10, 54, 61–63). Dysregulation of ACCs in diseases is therefore intensively studied.

## Dysregulation of Acetyl-CoA Carboxylases in Diseases

Fatty acid synthesis is central in the cross-talk between multiple biological processes, including membrane biosynthesis, energy storage, and the generation of signaling molecules (64). Lipogenesis is dynamically regulated in response to the physiologies of cells. Correspondingly, dysregulation of fatty acid synthesis can induce or promote the development of diseases. ACCs is the first rate-limiting enzyme in fatty acid synthesis. It is therefore the focus of mountains of studies and be validated as a critical participant in diseases, especially cancer and metabolic diseases (11, 65–71).

Signaling regulators of lipid biosynthesis are major downstream targets of oncogenes and tumour suppressor pathways. Alternations of oncogenes and tumour suppressor pathways can manipulate *de novo* fatty acid synthesis. Dysregulation of fatty acid metabolism, in turn, influences the cellular processes that are linked to diseases, such as cancer. For example, the AMPK pathway is important in regulating cell growth, lipid and glucose metabolism, and autophagy (72). It senses the relative level of ADP to ATP and be activated when the ratio of ADP to ATP increased. When tumour cells suffering metabolic stresses, AMPK can be activated, which then phosphorylates ACCs to suppress the lipid biosynthesis pathway, resulting in metabolism reprogramming that influences the survival and growth of tumour cells. Mutagenic blockage of the AMPK phosphorylation site of ACC (ACC1 Ser76Ala and ACC2 Ser212Ala) increases liver DNL and accelerates the development of hepatocellular carcinoma (HCC). Liver-specific inhibitor ND-654, which mimics ACC phosphorylation, inhibits liver DNL and the progression of HCC, resulting in an improved prognosis for tumour-bearing mice (73). In head and neck squamous cell carcinoma cells (HNSCC), the AMPK activator cetuximab and 5-aminoimidazole-4-carboxamide-1-β-D-ribofuranoside (AICAR) can suppress tumour cell growth (74, 75). Abolishing the AMPK phosphorylation sites on ACC1 by mutagenesis can protect HNSCC from cetuximab-induced growth inhibition. Decreased AMPK activity in hereditary leiomyomatosis renal cell cancer (HLRCC) leads to the elevated activity of ACC1, which contributes to the oncogenic growth of HLRCC (76). Metformin is an agonist of AMPK that can promote phosphorylation of ACCs. Metformin treatment can effectively suppress lipogenesis and cancer cell proliferation (10). Because ACC1 can mediate the AMPK-sensed metabolic stress and the downstream of cancer metabolism reprogramming, it is considered a potential target for cancer therapy. However, some studies also show exceptional viewpoints on the AMPK/ACC signaling pathway for tumour growth (77). For example, under energy stress conditions, activated AMPK can phosphorylate and inhibit ACC1, which can suppress the NADPH-consuming fatty acid synthesis and maintain the NADPH homeostasis in tumour cells. Similarly, ACC1 depletion can also suppress the NADPH consumption by fatty acid synthesis, which in turn partially facilitates solid tumour survival under stress conditions (77). Thus, under special conditions, the AMPK/ACC signaling pathway that can alternatively regulate tumour cell proliferation by maintaining NADPH homeostasis.

The phosphatidylinositol-3 kinase (PI3K)/Akt/mammalian target of the rapamycin (mTOR) is another signaling pathway that senses the physiologies of cells and executes important functions by regulating ACC1 activities. In general, receptor tyrosine kinases (RTKs)-mediated activation of PI3K can activate Akt. Hyperactivated Akt then activates mTOR, which processes the upstream signals and forms mTORC1 (78). PI3K/Akt/mTOR signaling pathway regulates tumour metabolism, growth, survival, and metastasis (79, 80). ACC1 is tightly associated with the PI3K/Akt/mTOR signaling pathway in cancer cells. For example, the melanoma antigen ganglioside GD3 is a downstream target of PI3K/Akt/mTOR signaling. In melanoma, GD3 can induce the activation of SREBP-1, which is a transcription factor that regulates the expression of ACC1 (81). In breast cancer, the HER2 oncogene can induce ACC1 expression through translational regulation of the mTOR signaling pathway (82). Correspondingly, inhibition of ACC1 by siRNA or chemical inhibitors can inhibit AKT-related pathways, which is detrimental for cancer, such as human HCC (83). It is therefore concluded that ACC1 protein level and activity can be regulated by various internal alterations, which in turn affects lipid synthesis in tumors. Dysregulated lipid metabolism impacts multiple intracellular processes, such as membrane synthesis and energy metabolism that may influence tumor development ultimately. However, the mechanisms underlying lipid metabolism influencing tumor progressions, such as proliferation and metastasis, have not been fully elucidated. How ACC1 cross-talk with other pathways remains open for discussion.

Metabolic diseases are also tightly associated with the dysregulation of ACCs. In mammals, the accumulation of lipid in tissues, such as muscle and liver, is closely related to insulin resistance that associates with a myriad of metabolic disorders (84, 85). Likewise, dysregulated lipogenesis may lead to metabolic diseases such as obesity, diabetes, and NAFLD (6–8). As a central player of lipogenesis, ACCs promptly participates in the progression of metabolic disease. For example, a high-fat diet leads to increased ACC1 activity and obesity in mice while inhibition of ACC1 antagonizes the high fat diet induced obesity. ACC2 plays roles in controlling diet-induced diabetic nephropathy (DN). High-glucose diets promote lipid deposition and reduce fatty acid β-oxidation in human podocytes. Depletion of ACC2 attenuates the high-glucose diet-promoted lipid deposition and podocyte injury. The expression of glucose transporter 4 (GLUT4) is also restored by ACC2 depletion, which hampers the insulin signaling pathway. Besides, the expression of SIRT1/PGC-1a, an important complex related to the insulin metabolic pathway is also restored in the cells with ACC2 depletion, leading to the reduction of cellular insulin resistance and ultimately alleviating DN-induced cell injury (86). ACCs knockout animal models are powerful tools to understand the roles of ACCs in the progression of metabolic diseases, with which, a study demonstrated that ACC1 is necessary to maintain functional pancreatic β cells and glucose homeostasis *in vivo*, which indicates that ACC1 might be used to improve insulin secretion during diabetes (71). Liver-specific ACC1-KO mice (LACC1 KO) accumulate 40%-70% lower triglycerides in livers than that of wide-type mice when overnutrition is provided. Similarly, ACC2 knockout (ACC2 KO) mice do not gain weight when fed with high-fat diet (HF) (34). It might be due to the hepatic peroxisome proliferator-activated receptor-γ (PPAR-γ) proteins that are significantly reduced in ACC2 KO mice that are fed with high-fat and high-carbohydrate diet (HFHC). In this case, lipid synthesis-related enzymes such as ACC1, FASN, and ATP citrate lyase (ACL) are decreased, which in turn reduced diet-induced obesity. Besides, ACC2 KO mice are able to alleviate the HFHC diet-induced insulin resistance. ACC2 KO mice with HF diet show reduced AKT level and increased phosphorylation of AKT, which is critical in the insulin signaling pathway that can protect the mice from diabetes (87). The above researches demonstrate that ACCs is responsible for metabolic disorders caused by dietary factors (27). Moreover, hyper-activation of ACC1 can also result in abnormal physiologies in metabolic disease. For instance, the enhanced activity of ACC1 accelerates lipogenesis and lipid accumulation when animal suffering overnutrition and obesity, which leads to the accumulation of triglycerides in hepatocytes and thus causing NAFLD (88).

In general, dysregulated lipogenesis leads to the development of tumorigenesis and metabolic diseases. The roles of ACCs in regulating metabolism reprogramming in cancer and metabolic diseases are revealed in accumulated studies, which shed bright light on diseases treatment. As such, ACCs are becoming a promising therapeutic target for discovering novel therapeutic strategies and therapeutics development.

## Acetyl-CoA Carboxylases-Targeting Small Molecules for Therapeutic Proposes

With the evidence of ACCs participating in the progression of diseases and its structural information, countless screenings for ACCs antagonists are performed and several promising leading compounds are confirmed for further validations (65, 83, 89–94). The ACCs inhibitors mainly target its BC domain and CT domain.

The BC domain accounts for the biotin carboxylation and formation of the homodimer of ACCs molecules. The main mechanism of action (MOA) of the BC domain targeting inhibitors is allosterically inhibiting the dimerization of the BC domain, maintaining ACC1 molecules as inactive monomers that are unable to perform the catalytic activity (21). Soraphen A, AMPK activators, and ND-series inhibitors (ND-630, ND-646, ND-654) inhibit ACC1 belong to this category (73, 91, 95–98). These inhibitors can effectively inhibit ACCs activity and affect the process of lipid metabolism and the development of disease (10, 26, 28, 29, 90, 99, 100). It is worth noting that there are studies already confirmed that inhibiting ACCs in the liver by using ND-630 (GS-0976) can significantly reduce 29% liver fat, hepatic steatosis, and markers of liver injury in NAFLD patients (101, 102), which further encourage the finding of ACCs inhibitors for therapeutic proposes.

The CT domain catalyzes the transfer of carboxyl-moiety from the carboxylated biotin to the acetyl-CoA to produce malonyl-CoA. Competitive inhibitors targeting the binding of acetyl-CoA by CT domain are therefore another promising strategy for inhibiting ACCs. TOFA, CP-640186, piperidinyl derived analogs, and spiropiperidine derived compounds are antagonists that belong to this category (103–108). These antagonists can reduce the mice’s appetite and accelerate weight loss (93, 109) and lead to apoptosis in different cancer cell lines (92, 110, 111). Despite no relevant clinical trials of this class of antagonists are found, it keeps recruiting screenings for new leading compounds.

In conclusion, numbers of commercially available ACCs inhibitors have exhibited strong therapeutic effects on disease models *in vivo* and *in vitro*, supporting that ACCs are promising therapeutic targets for the treatment of tumour and metabolic diseases. However, no agonist can specifically inhibit one ACCs member and keep another member intact. This might lead to adverse effects, because ACC1 and ACC2, indeed, are different in physiologies and pathophysiologies. To this end, the development of agonists that are specifically against ACC1 or ACC2 might be a promising strategy to target ACCs for diseases treatment.

## Limitations and Prospects

Antagonists that target ACCs are intensively studied in clinic but hampered by several side-effects. For example, inhibiting lipogenesis *via* suppressing the expression of ACCs can reduce hepatic steatosis, but it simultaneously results in hypertriglyceridemia due to the activation of SREBP-1c and increased VLDL secretion (40). Another instance is PF-05175157, the first-in-human clinical trials ACC inhibitor, contributes to DNL reduction in treatment for T2DM but with concomitant reductions in platelet count (112). Recently, an exciting result in phase II clinical trial shows that the co-administration of PF-0522134 (a new ACC1 inhibitor in clinical trial) and PF-6865571 (DGAT2 inhibitor) has a strong effect in treating NASH without the side effect of hypertriglyceridemia (113). However, there are several challenges to address the side-effects of ACCs inhibition in clinical practice.

The principal challenge is that the inhibitors can hardly distinguish ACC1 from ACC2. As described above, ACC1 and ACC2 share 75% identity in amino acid sequence and are similar in structures that are composed of conservative domains for enzyme activity. However, the ACCs antagonists, such as Soraphen A and TOFA, can target and influence the activity of both ACC1 and ACC2, which might lead to the side-effects caused by the inhibition of unwanted ACC isoform. In nutrient-abundant condition, fatty acid synthesis and breakdown are coordinately controlled, avoiding a wasteful cycle of metabolism. However, in cancer cells, both fatty acid synthesis and breakdown are boosted to support cancer growth. To this end, coordinately antagonizing the dysregulation of ACC1 and ACC2 in cancer cells would be a promising strategy for cancer treatment. So far, none of ACCs inhibitors is approved useful in clinic. This might be due to the fact that ACC inhibitors that are not isoform-specific only partially reverse cancer’s preferences. Moreover, it is shown that the selectively inhibition of ACC2 may be ineffective in treating some metabolic diseases (114). A selective inhibitor targeting ACC1 that shows anti-NAFLD/NASH effects in pre-clinical models is reported in a recent study (115), which is expected to strengthen the efficacy.

Accumulating studies indicate the importance of ACCs in tumour cell growth which shows the great potential of ACCs in the treatment of cancer. However, studies on the role of ACCs in cancer have been attributed to their roles in fatty acid synthesis, the exact mechanism of which remains to be investigated. The role of fatty acid metabolism in cancer biology is not fully understood (116). More in-depth research about fatty acid metabolism in cancer will help examine and detail the roles of the ACCs, in cancer initiation, progression, and development.

## Author Contributions

YuW: draft the manuscript and iconography. WY, SL, DG, and JH: proofread the manuscript and iconography. YugW: conceptualized, supervised, and finalized the manuscript. All authors contributed to the article and approved the submitted version.

## Funding

This work was supported by the National Natural Science Foundation of China (31970577 and 91957110).

## Conflict of Interest

The authors declare that the research was conducted in the absence of any commercial or financial relationships that could be construed as a potential conflict of interest.

## Publisher’s Note

All claims expressed in this article are solely those of the authors and do not necessarily represent those of their affiliated organizations, or those of the publisher, the editors and the reviewers. Any product that may be evaluated in this article, or claim that may be made by its manufacturer, is not guaranteed or endorsed by the publisher.
